# Dietary supplementation of β-conglycinin, with or without sodium butyrate on the growth, immune response and intestinal health of hybrid grouper

**DOI:** 10.1038/s41598-021-96693-x

**Published:** 2021-08-27

**Authors:** Bin Yin, Hongyu Liu, Beiping Tan, Xiaohui Dong, Shuyan Chi, Qihui Yang, Shuang Zhang

**Affiliations:** 1 Laboratory of Aquatic Animal Nutrition and Feed, Fisheries College, Guangdong Ocean University, Zhanjiang, 524025 People’s Republic of China; 2 Aquatic Animals Precision Nutrition and High Efficiency Feed Engineering Research Centre of Guangdong Province, Zhanjiang, Guangdong People’s Republic of China; 3 Key Laboratory of Aquatic, Livestock and Poultry Feed Science and Technology in South China, Ministry of Agriculture, Zhanjiang, 524025 People’s Republic of China

**Keywords:** Antigen processing and presentation, Inflammation, Ichthyology

## Abstract

We investigated the effects of low and high doses of β-conglycinin and the ameliorative effects of sodium butyrate (based on high-dose β-conglycinin) on the growth performance, serum immunity, distal intestinal histopathology, and gene, protein expression related to intestinal health in hybrid grouper (*Epinephelus fuscoguttatus* ♀ × *E. lanceolatus* ♂). The results revealed that the instantaneous growth rate (IGR) of grouper significantly increased, decreased, and increased in the low-dose β-conglycinin (bL), high-level β-conglycinin (bH) and high-level β-conglycinin plus sodium butyrate (bH-NaB), respectively. The feed coefficient ratio (FCR) was significantly increased in the bH and bH-NaB, serum levels of IFN-γ, IL-1β, and TNF-α were upregulated in the bH. The intestinal diameter/fold height ratio was significantly increased in the bH. Furthermore, there were increases in nitric oxide (NO), total nitric oxide synthase (total NOS), and peroxynitrite anion (ONOO^−^) in the bH, and decreases in total NOS and ONOO^−^ in the bH-NaB. In the distal intestine, IL-1β and TGF-β1 mRNA levels were downregulated and upregulated, respective in the bL. The mRNA levels of TNF-α and IL-6 were upregulated in the bH, and downregulated in the bH-NaB, respectively. Occludin, claudin3 and ZO-3 mRNA levels were upregulated in the bL, downregulated in the bH and then upregulated in the bH-NaB. No significant differences were observed in the mRNA levels of IFN-γ and jam4. And the p-PI3K p85^Tyr458^/total PI3K p85 value was significantly increased in the bH and then decreased in the bH-NaB, and the total Akt value was significantly increased in the bH. These indicate β-conglycinin has a regulatory effect on serum immunity and affect distal intestinal development by modulating distal intestinal injury-related parameters. Within the distal intestinal tract, low- and high-dose β-conglycinin differentially affect immune responses and tight junctions in the distal intestine, which eventually manifests as a reduction in growth performance. Supplementing feed with sodium butyrate might represent an effective approach for enhancing serum immunity, and protects the intestines from damage caused by high-dose β-conglycinin.

## Introduction

The hybrid grouper (*Epinephelus fuscoguttatus ♀* × *E. lanceolatus ♂*) is characterized by a rapid growth rate and desirable meat quality, which is of high economic value. As a typical carnivorous marine fish, hybrid grouper typically require up to 50–55% protein in their feed^1^, which is generally provided by fishmeal containing high levels of nitrogen and phosphorus. In addition, soybean meal is also a widely available and inexpensive source of protein for hybrid grouper diets. However, hybrid grouper have a low tolerance to soybean meal, which can make up approximately 40–60% of fishmeal diets^2^. We speculate that this is related to the presence of immunogenic β-conglycinin (7S) in soybean meal^3–5^. β-conglycinin accounts for approximately 30% of the soy protein content^6^. Similar to other proteins, 7S is absorbed by animals and decomposed into small peptides and amino acids, but a small portion of 7S would enter the body's lymphatic system directly through the intestinal mucosa, thus causing adverse reactions in the body^7^. In piglets, 7S allergic reactions mainly induce a reduction in the height of the intestinal villi and thereby reduces the efficiency of nutrient absorption^8^, whereas in grass carp, 7S can promote an increase in reactive oxygen species (ROS) production in the distal intestine, leading to disruption of the structural integrity of epithelial cells^3^. However, the mechanisms underlying the intestinal tolerance to 7S and the intestinal inflammation caused by this compound in hybrid grouper have yet to be determined.

Short-chain fatty acids include acetic acid, propionic acid, and butyric acid, with the latter playing a major role in regulating the organism metabolism^9^. It has been established that the hydrolysis of sodium butyrate (NaB: the sodium salt form of butyric acid) to butyric acid has a wide range of physiological effects, providing an energy source for intestinal cells, promoting intestinal cell differentiation and proliferation, and maintaining intestinal morphology to facilitate digestion and absorption^10,11^. Accordingly, given its unique nutritional and physiological functions and lack of polluting and harmful residues, sodium butyrate has become a valuable additive that is used to supplement the diets of cultured aquatic animals.

The intestinal tract of fish is well supplied by nerves and microvasculature, and is often referred to as the second brain of the fish. The intestinal immune barrier consisting of intestinal mucosal epithelium is an important defensive line against pathogenic invasion^12^. Accordingly, as the most fully functional immune organ in fish, the intestine is directly affected by antigenic proteins from the feed and produces the corresponding immune response^3^. Unlike mammals, the uptake and deposition of 7S occurs mainly in the distal intestine (about 20–25% of the total intestine)^13,14^. Meanwhile, the distal intestine contains macrophages, lymphocytes, etc., and also has abundant swallowing vesicles and mitochondria^15^. At present, it is unknown whether 7S-induced intestinal inflammation in fish is associated with the very small fraction of 7S that is not hydrolyzed and enters the lymphoid tissue through the epithelial cell gap^16^. The absorption of excessive large molecular antigenic proteins in the fish intestine usually leads to intestinal inflammation, a process that is closely linked to the changes in cytokines, like IFN-γ, TNF-α and TGF-β^17^, exhibiting low nutrient absorption efficiency and high permeability. Likewise, this invasion can also negatively affect the intestinal barrier, mainly by disrupting the tight junctions of the intestine^18,19^. Research in our laboratory has revealed that intestinal inflammation in hybrid grouper caused by ingesting feed containing high levels of soybean meal is related to an abnormal activation of the PI3K/Akt signaling pathway^20^. Furthermore, it has also been found that sodium butyrate can significantly inhibit the Akt/mTOR signaling pathway^21^, and thus we speculate that by inhibiting PI3K/Akt/mTOR, sodium butyrate could be used to relieve the intestinal inflammation caused by 7S.

In this study, we therefore used hybrid grouper as experimental animals to evaluate the hypothesis that dietary β-conglycinin might affect growth by regulating serum immunity, distal intestinal histopathology, epithelial injury, and the PI3K/Akt signaling pathway. In addition, we also investigated the potential protective effects of appropriate doses of sodium butyrate against high levels of dietary 7S. We anticipate that the findings of this study will provide a reference for research on improving the tolerance of hybrid grouper to soybean meal and reveal the mechanisms underlying the protective effects of sodium butyrate on the distal intestine.

## Methods

The animal protocol used in the present study was approved by the ethics review board of Guangdong Ocean University, and the study was carried out in compliance with the ARRIVE guidelines (The ARRIVE guidelines 2.0). All methods were carried out in accordance with relevant guidelines and regulations.

### Feeding trial and challenge test

The juvenile hybrid grouper used in this study were purchased from a commercial hatchery on East Island (Zhanjiang, China), and were derived from the same batch of artificially hatched fish. Prior to experimentation, the fish were acclimated to the local environment by placing in an outdoor pond of the Biological Research Base of Guangdong Ocean University for 1 week, during which time they were provisioned with commercial feed.

Having acclimated, 480 healthy uniformly sized hybrid groupers (initial body weight: 7.70 ± 0.05 g) were selected and randomly divided into four groups, each with four replicates of 30 fish, and the fish were cultured in 300-L glass fiber buckets. During the culture period, the fish were fed twice daily (at 08:00 and 16:00) to apparent satiety with a water change of approximately 70%. Dissolved oxygen levels in the containers were maintained at ≥ 7.00 mg/L via continuous aeration using air stones, whereas water temperature, pH, and ammonia nitrogen content were maintained at 29.00 ± 1.30 °C, 7.8–8.1, and < 0.09 mg/L, respectively. At the end of the 8-week culture period, fish fed the same diet and under the same culture conditions as the FM group were used for pre-experiments, where they were injected with different concentrations and doses of the bacterial solution to obtain median lethal concentrations (data not shown). After that, 60 fish were randomly selected for each treatment, 40 (10 fish per replicate) of which were used for the challenge test, and the other 20 (5 fish per replicate) were used as blank test. For challenge test, each fish was injected intraperitoneally with 200 µL of *Vibrio parahaemolyticus* at a concentration of 7.41 × 10^8^ CFU/mL, and the same dose of sterilized PBS for blank test, and thereafter the fish were continuously observed for 1 week, with mortality being recorded daily.

### Diet formulations

Experimental fish were provided with one four diets containing protein and fat at levels of approximately 48% and 10%, respectively, which were prepared using redfish meal, casein, and gelatin as the main protein sources, and fish oil and soy lecithin as the main lipid sources. As treatments, the diet was supplanted as follows: 0% 7S, 0% NaB (fishmeal group, FM); 1.50% 7S (low-dose^22^ 7S, bL), 0% NaB; 6.00% 7S, 0% NaB (high-level 7S, bH); 6.00% 7S, 0.13% NaB (high-level 7S with NaB, bH-NaB) (Supplementary Table S1). The three experimental diets were also supplemented with methionine and lysine to same level as that in the FM diet. The essential amino acid profiles of the diets used in this experiment are shown in Supplementary Table S2. Purified 7S was purchased from the China Agricultural University (Patent No. 200410029589.4, China). The SDS-PAGE profile of purified 7S fraction was shown in Supplementary Fig. S1. For use in the experimental diets, water was added to the purified β-conglycinin several times in small amounts, stirred thoroughly, and the pH of the solution was adjusted to 7.2. The 7S solution was then placed in a glass petri dish and dried for 72 h in a low-temperature freeze dryer. After crushing the different raw materials of the diet through 380-μm meshes, the components mixed in a stepwise manner, adding fish oil, soybean lecithin, and 30% water and mixing evenly. The resulting mixture was then placed into a feed pelletizer, which produced feed pellets of 2.5 mm in diameter. The pellets were air-dried at room temperature until the moisture content was approximately 10% (approx. 48 h), sealed in a self-sealing bag, and stored at − 20 °C until required. The 30% microencapsulated sodium butyrate used in this study was kindly provided by Shanghai Menon Animal Nutrition Technology Co., Ltd.

### Sample collection

After the end of the 8-week breeding experiment, the fish in each bucket were fasted for 24 h and then counted and weighed. For each replicate, two fish were randomly selected, from which blood was drawn using a 1 mL syringe. After being left to stand for 12 h, the blood samples were centrifuged for 10 min at 5000 r/min and 4 °C. The serum thus obtained was collected and stored at − 80 °C for the determination of serum immune indicators. A further six fish were randomly selected for each replicate, from which the intestines were gently removed, and one third of the whole intestine was taken as distal intestine (DI) near the cloaca. Two of the DI segments for each replicate were placed in 4% formaldehyde solution for the preparation of Alcian Blue-Periodic acid-Shiff (AB-PAS) staining and TUNEL staining, and a further two DI segments were placed in liquid nitrogen for the determination of distal intestine-related enzyme activities and protein expression. After the DI tissue had been thawed on ice, dried with qualitative filter paper, and weighed, a sample was homogenized in homogenization medium (0.8% sodium chloride solution) in an ice bath at a sample: medium ratio of 1:9 (mass to volume ratio), centrifuged at 5000 r/min for 10 min at 4 °C, and the supernatant was collected for analysis. The remaining two DI samples from each replicate were placed in RNA latter, and after being left to stand for 12 h, were stored in a freezer at − 80 °C for subsequent determination of related gene expression.

### Determination of growth and DI evaluation indicators

Parameters of interest were determined as follows:

$${\text{WG }}\left( {{\text{weight}}\,{\text{ gain}}, \, \% } \right) \, = { 1}00 \, \times \, \left( {{\text{final }}\,{\text{weight }} - {\text{ initial}}\,{\text{ weight}}} \right)/{\text{initial}}\,{\text{ weight,}}$$

$${\text{IGR }}\left( {{\text{instantaneous }}\,{\text{growth }}\,{\text{rate}}, \, \% /{\text{day}}} \right) \, = { 1}00 \, \times \, \left[ {{\text{ln}}\left( {{\text{final}}\,{\text{ weight}}} \right) \, - {\text{ ln}}\left( {{\text{initial }}\,{\text{weight}}} \right)} \right]/{\text{days}}\,{\text{ of }}\,{\text{experiment,}}$$

$${\text{SR }}\left( {{\text{survival }}\,\% } \right) \, = {1}00 \, \times {\text{final }}\,{\text{fish }}\,{\text{number}}/{\text{initial}}\,{\text{ fish }}\,{\text{number,}}$$

$${\text{FCR }}\left( {{\text{feed }}\,{\text{coefficient }}\,{\text{ratio}}} \right) \, = {\text{ dry}}\,{\text{ feed }}\,{\text{consumed}}/{\text{weight }}\,{\text{gain,}}$$

$${\text{Id}}/{\text{Fh }} = {\text{ intestinal}}\,{\text{ diameter}}/{\text{fold }}\,{\text{height}}{.}$$

### AB-PAS staining and TUNEL staining determinations

After the tissue was removed from 4% formalin buffer, it was paraffin-embedded and cut into 4 μm sections using a microtome (Leica Instruments Shanghai, RM2016). Dewax the sections by Xylene I for 20 min, Xylene II for 20 min, 100% ethanol I (Servicebio, G1049) for 5 min, 100% ethanol II for 5 min and 75% ethanol for 5 min, then rinsed with tap water. After that, for AB-PAS, stained sections with Alician blue dyes for 15 min, rinsed with tap water until colorless. Then Stain with periodic acid dye for 15 min, rinsing with tap water, and rinsed twice with distilled water. Brought Schiff reagent to room temperature, and stained with it for 30 min in the dark, rinsed for 5 min. At last, the sections were dehydrated by 100% ethanol I for 5 min, 100% ethanol II for 5 min, 100% ethanol III for 5 min, Xylene I for 5 min, Xylene II for 5 min, and sealed with neutral gum. Orthomorphic fluorescence microscopes (Nikon SMZ 18), with a camera (Nikon DS-Ri2), a 4 × objective lense (CFI, Plan Fluor, N.A. 0.13, W. D. 17.2 mm) and 20 × object lense (CFI, Plan Fluor, N.A. 0.50, W. D. 2.1 mm), were used to make observations on sections. Section images were acquired under 10 × eyepiece, 4 × objective and 10 × eyepiece, 20 × objective, respectively. Ten folds were randomly chosen for data analysis. Fold height, intestinal diameter and goblet cells were measured and observed by cellSens Standard 1.8 and LAS 3.8 software. For TUNEL staining, eliminated obvious liquid, marked the objective tissue with liquid blocker pen. Added proteinase K working solution to cover objectives and incubate at 37 °C for 25 min, then washed three times with PBS (pH 7.4), 5 min each. The tissue was covered using the permeabilize working solution (0.1% triton, triton stock solution: PBS = 1:1000), incubated for 30 min at room temperature, and washed 3 times again using PBS, 5 min each. Then added equilibration buffer and incubated at room temperature for 10 min. Mixed TDT enzyme, dUTP and buffer in the ratio of 1:5:50, covered the tissue and incubated for 2 h at 37 °C. After washing three times with PBS, added DAPI staining solution and incubated for 10 min at room temperature avoiding light, washed three times with PBS, and threw away liquid slightly, then covered slip with anti-fade mounting medium. Microscopic examination and collecting images through orthomorphic fluorescence microscopes (Nikon Eclipse C1), with a camera (Nikon Eclipse Ci-L), and 40 × object lense (CFI, Plan Fluor, N.A. 0.75, W. D. 0.66 mm).

### Immune index determinations and distal intestinal NO, ONOO^−^ and total NOS determination

Serum immune and DI injury indices were determined using Shanghai Enzyme-Linked ELISA immunoassay kits (Full-wavelength microplate reader-1510). During the detection, the serum was diluted 5 times according to the instructions using the sample dilution solution to meet the kit assay range. Intestinal tissue preserved in liquid nitrogen was homogenized to obtain a tissue solution with a tissue mass (g)/[tissue mass with saline volume (mL)] of 20%. Centrifuged at 2500 rpm for 10 min and 0.16 mL and 0.1 mL of supernatant was taken as the sample for the determination of NO and total NOS, respectively.

Responding to tissue ONOO^−^ levels by measuring 3-NT level. The 3-NT antibody (SC-32731) was coated to a microtiter plate to make a solid phase antibody. The standards were diluted to 15 U/L, 30 U/L, 60 U/L, 120 U/L and 180 U/L, and 40 µL of diluent was added to each sample well before adding 10 µL of sample to the sample wells. After the plate is closed, incubate at 37° for 30 min, discard the liquid inside the wells and wash the plate with washing solution, gently shake the plate and pour off the washing solution, repeat 5 times. After cleaning the enzyme plate, added 50 µL each of chromogenic agent A and chromogenic agent B to each well, shaken and mixed gently, and developed the color for 15 min at 37 °C, protected from light. Then added 50 µL of termination solution to each well to terminate the reaction. After the color development reaction was carried out, the termination solution was added, the blank wells were zeroed, and the OD value was measured at 450 nm.

Experimental procedures were performed according to the instructions of the NO determination kit (#A012-0-2) and total NOS determination kit (#A014-2) provided by Nanjing Jiancheng Bioengineering Institute. The sodium nitrite standard solution was diluted into different gradients using double distilled water and used as a standard curve. Equal volumes of double-distilled water, sodium nitrite standard solution and serum samples were added to the blank, standard and assay wells, respectively. All wells were added with color developer and left for 15 min to determine the OD value at 550 nm. For total NOS, equal volumes of double-distilled water and sample were added to the blank and assay wells, respectively. All wells were then successively added with substrate buffer, promoter and color developer. 15 min of water bath at 37°, followed by the addition of clearing agent and termination solution. Double distilled water was zeroed, wavelength 530 nm, 1 cm optical diameter to determine the absorbance value of each tube.

### RT-PCR determination

Total RNA from hybrid grouper DI was extracted using a TranZol Up Plus RNA kit (Beijing TransGen Biotechnology Co., Ltd) in accordance with the manufacturer’s instructions. The integrity of the extracted RNA was assessed by 1% agarose gel electrophoresis, and a NanoDrop ND-2000 spectrophotometer (Thermo Scientific, USA) was used to determine the purity and concentration of RNA. RNA samples of sufficient quality should run as a clear and complete band, and have an OD_260_/OD_280_ ratio of between 1.8 and 2.0. A PrimerScript RT-PCR Kit (TaKaRa, Kusatsu, Japan) was used to reverse transcribe total RNA to cDNA, which was stored at − 20 °C prior to RT-PCR analysis. Specific primers for RT-PCR (Supplementary Table S3) were designed using Primer Premier 5.0 software based on full-length transcriptome sequences of hybrid grouper in the NCBI Sequence Read Archive with accession number PRJNA664416. All the real-time PCR reactions were run in an Applied Biosystems 7500 Real-Time PCR System (Life Technologies, Carlsbad, CA, USA) using a SYBR Premix Ex Taq Kit (Takara). The β-actin gene was used as an internal reference to normalize the amplification efficiency of each target gene, and the mRNA expression levels of genes were compared and analyzed using 2^−ΔΔCT^ methods according to Livak et al.^23^.

### Western blot determination

The protein supernatant was extracted from intestinal tissues. Protein concentration was determined using the BCA method, supplemented with PBS and loading buffer to adjust the final protein concentration of the sample to 2 µg/µL, and boiled in boiling water for 10 min. and finally quickly put the sample on ice, moved to − 80 °C refrigerator until used. SDS-PAGE was used to separate the protein samples (10 µL sample per lane). The steps of electrophoresis, membrane transfer, closure and antibody incubation are performed according to the previous methods in our lab^20^. The following antibodies were used in this study: antibodies against PI3 Kinase p85 (4292), phospho-PI3 Kinase (Tyr458, 4228S), Akt (9272S), phospho-Akt (Ser473, 9271S) and GAPDH (2118S) were all purchased from Cell Signaling Technology. On account of mammalian antibodies being used, amino acid sequences of studied protein from hybrid grouper were aligned in the NCBI database (https://blast.ncbi.nlm.nih.gov/Blast.cgi) to check the identity of the antibodies. The western bands were quantified using NIH Image 1.63 software.

### Statistical analyses

Survival between the experimental group and the control group were identified by the Kaplan–Meier plot Log-Rank (Mantel-Cox) test^24^. All data were subjected to one-way analysis of variance followed by Tukey multiple range tests to determine significant differences among treatment groups using SPSS v. 22 (IBM, USA) at a significance level of *P* < 0.05, as described by Guo et al.^25^. The results are presented as the means ± SE.

## Results

### Growth performance and challenge test

The results obtained for the growth performance of fish in different groups are presented in Table 1. These results indicated that compared with the FM group, the final body weight (FBW), weight gain (WG), and instantaneous growth rate (IGR) were all significantly higher in the bL group and lower in the bH group, whereas no significant differences were observed between the FM and bH-NaB groups. The feed coefficient ratio (FCR) in the bH group was found to differ significantly from that in the other three groups, although there was no significant difference between the FM and bL groups. In contrast, we detected no significant differences among the four groups with respect to survival (SR).

**Table 1:** Growth parameters and feed utilization of juvenile hybrid grouper fed the experimental diets for 8 weeks.

	FM	bL	bH	bH-NaB
IBW (g)	7.70 ± 0.05	7.72 ± 0.06	7.70 ± 0.04	7.69 ± 0.05
FBW (g)	50.60 ± 0.26^b^	56.21 ± 2.71^c^	42.09 ± 6.52^a^	51.11 ± 1.31^b^
WG (%)	557.22 ± 3.33^b^	636.50 ± 11.37^c^	464.18 ± 6.76^a^	579.87 ± 20.79^bc^
IGR (%/day)	3.36 ± 0.01^b^	3.57 ± 0.03^c^	3.09 ± 0.02^a^	3.42 ± 0.05^bc^
SR (%)	100.00 ± 0.00	100.00 ± 0.00	99.17 ± 0.83	100.00 ± 0.00
FCR	0.81 ± 0.05^a^	0.77 ± 0.05^a^	1.52 ± 0.21^c^	1.04 ± 0.08^b^

Value show means ± SE (n = 4); Significance was evaluated by one-way ANOVA followed by Tukey’s multiple range tests.

*FM* control diet, *bL* containing 1.5% 7S diet, *bH* containing 6% 7S diet, *bH-NaB* containing 6% 7S and 0.13% NaB diet, *IBW* initial body weight, *FBW* final body weight, *WG* weight gain, *IGR* instantaneous growth rate, *SR* survival, *FCR* feed coefficient ratio.

^a,b,c,d^Mean values among all treatments with different letters were significantly different when the interaction was significant (*P* < 0.05).

**Figure 1:**
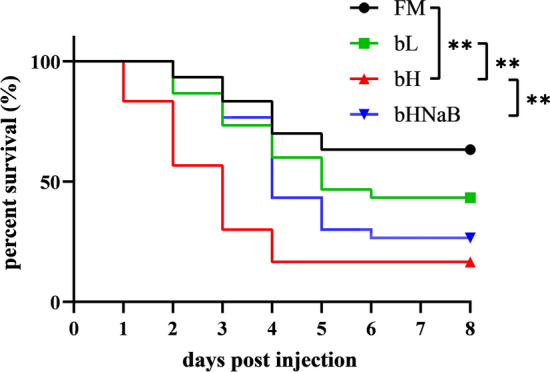
Cumulative survival against *Vibrio parahaemolyticus* of juvenile hybrid grouper after feeding with the four experimental diets for 8 weeks. Mortality was measured in each treatment groups (n = 40) and was recorded every 12 h post-injection (*indicates *P* < 0.05 and **indicates *P* < 0.01). Median survival was undefined in the FM (black), 5 days in the bL (green), 3 days in the bH (red) and 4 days in the bHNaB (blue). The Chi square values of each comparison objects are 2.216 for FM vs bL, 18.89 for FM vs bH, 10.17 for bL vs bH and 7.303 for bH vs bHNaB.

The cumulative mortality of fish in response to injecting with *V. parahaemolyticus* is shown in Fig. 1, which indicates that fish in the bH and bH-NaB groups suffered heavier mortalities than those in the FM and bL groups, with all groups reaching an asymptote at approximately 5 days post-injection. Among the different groups, the bH group showed a sustained and rapid increase in cumulative mortality from the first day post-injection, whereas in the bHNaB group, mortality increased slowly over the first 3 days after injection, although thereafter showed a rapid increase. As for the blank control groups of the four groups, no mortality occurred and the survival were 100% in all cases.

### Serum immune indices

**Figure 2:**
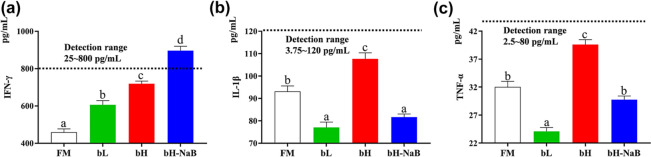
Serum immune indexes of juvenile hybrid grouper fed experimental diets for 8 weeks. Values are the means ± SE (n = 4); Significance was evaluated by one-way ANOVA followed by Tukey multiple range tests. *FM* control diet, *bL* containing 1.5% 7S diet, *bH* containing 6% 7S diet, *bH-NaB* containing 6% 7S and 0.13% NaB diet. Dashed horizontal line: detection limit. (**a**) IFN-γ, interferon-gamma; (**b**) IL-1β, interleukin-1 beta; (**c**) TNF-α, tumor necrosis factor-alpha. ^a,b,c,d^Mean values denoted by different superscripted letters differ significantly at the *P* < 0.05 level.

The serum immune indices determined for the different groups are presented in Fig. 2. Compared with the FM group, we detected significant increases in serum IFN-γ in the bL, bH, and bH-NaB groups, among which the bH-NaB group showed the most significant difference. The levels of IL-1β were found to be significantly increased in the bH group and significantly reduced in the bL and bH-NaB groups, although no significant differences were detected between the bL and bH-NaB groups. With respect to TNF-α, we observed significantly downregulated and upregulated levels in the bL and bH groups, respectively, compared with the FM group, whereas in contrast, there was no significant difference between the bH-NaB and FM groups.

### Distal intestinal histopathological evaluation and damage indicators

**Figure 3:**
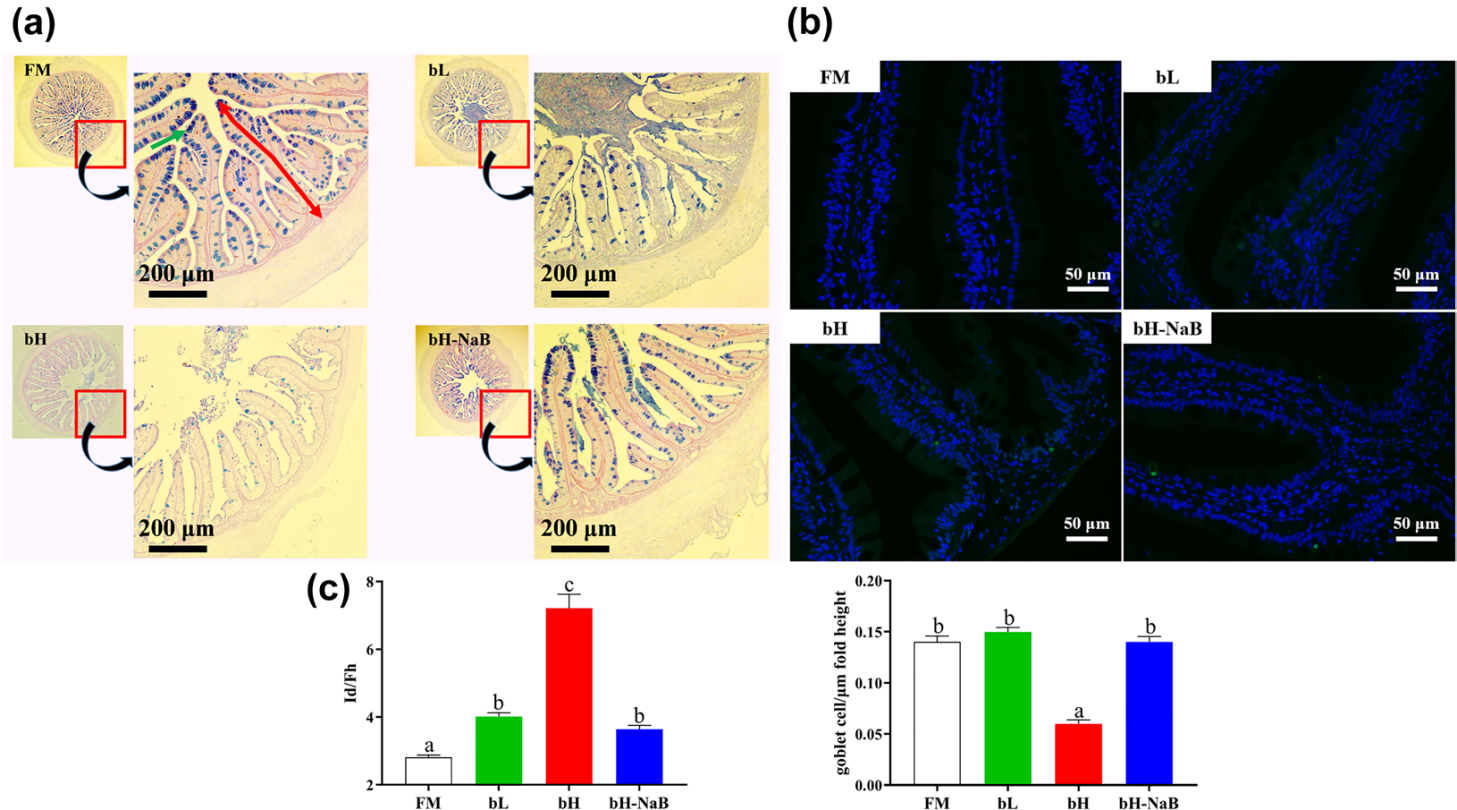
Distal intestinal histological evaluation based Alcian Blue-Periodic acid-Shiff staining (**a**) and TUNEL staining (**b**) of distal intestine sections obtained from juvenile hybrid grouper fed experimental diets for 8 weeks. Green arrow: goblet cell; red arrow: fold height. Results are represented as the means ± SE (n = 8) (**c**). Significance was evaluated by one-way ANOVA followed by Tukey multiple range tests. *FM* control diet, *bL* containing 1.5% 7S diet, *bH* containing 6% 7S diet, *bH-NaB* containing 6% 7S and 0.13% NaB diet. ^a,b,c^Mean values denoted by different superscripted letters differ significantly at the *P* < 0.05 level (*P* < 0.05).

**Figure 4:**
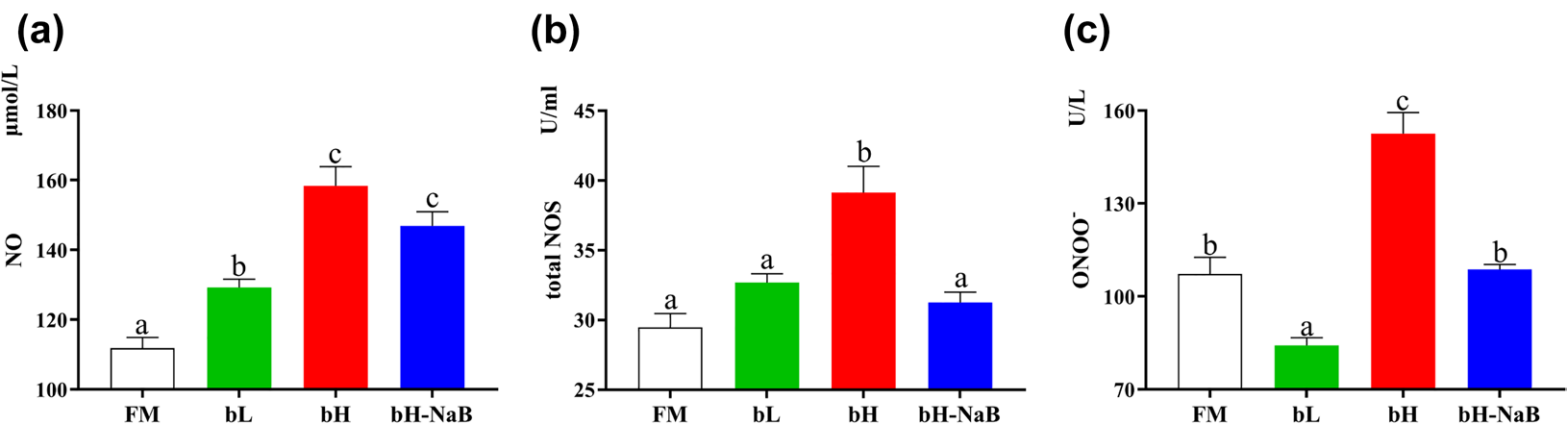
Distal intestinal damage indicators of juvenile hybrid grouper fed the experimental diets for 8 weeks (*P* < 0.05). (**a**) NO, nitric oxide; (**b**) total NOS, total nitric oxide synthase; (**c**) ONOO^−^, peroxynitrite. ^a,b,c^Mean values denoted by different superscripted letters differ significantly at the *P* < 0.05 level.

The results obtained for the different groups with respect to distal intestinal histopathology evaluated by AB-PAS staining, TUNEL staining, developmental status assessment, and damage indicators determined are presented in Figs. 3 and 4, respectively. As shown in Fig. 3, the intestinal diameter/fold height ratios (Id/Fh) obtained for the bL and bH-NaB groups were significantly higher than that for the FM group, and significantly lower than that for the bH group. Among the four groups, the bH group was found to have the lowest density of mucus cells, whereas we detected no significant differences among the other three groups. In addition, no apoptotic cells were found in the FM and bL, and only two apoptotic cells were found in each of the bH and bHNaB.

Compared with the FM group, the levels of nitric oxide (NO) were significantly upregulated in the bH and bH-NaB groups (Fig. 4). Similarly, although the levels of NO in the bL groups were somewhat lower, they were still significantly higher than those in the FM group. Whereas we observed that levels of nitric oxide synthase (NOS) in the bH group were significantly higher than those in the FM, bL, and bH-NaB groups, we detected no significant difference among the FM, bL, and bH-NaB groups. Moreover, levels of the peroxynitrite anion (ONOO^−^) were found to be lowest in the bL group, whereas no significant differences were observed among the FM, bH, and bH-NaB groups.

### Distal intestinal immune-, tight junction-related genes expression and protein expression

**Figure 5:**
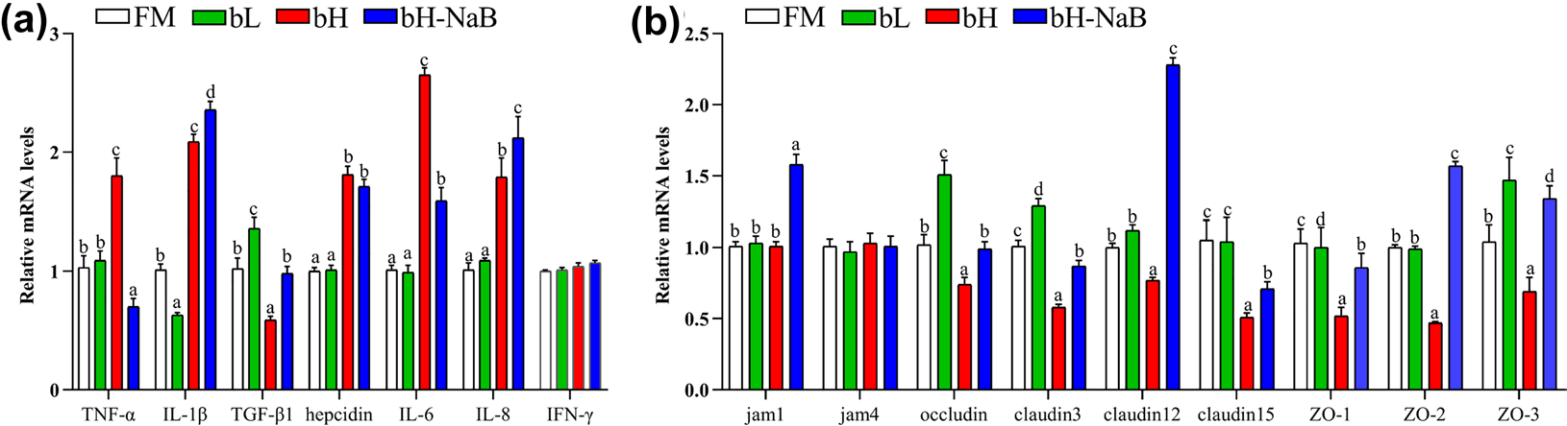
Distal intestinal inflammation- and tight junctions-related genes expression in juvenile hybrid groupers (*Epinephelus fuscoguttatus* ♀ × *E. lanceolatus* ♂) fed the experimental diets for 8 weeks. Results are represented as the means ± SE (n = 8). Significance was evaluated by one-way ANOVA followed by Tukey multiple range tests. *FM* control diet, *bL* containing 1.5% 7S diet, *bH* containing 6% 7S diet, *bH-NaB* containing 6% 7S and 0.13% NaB diet. ^a,b,c,d^Mean values denoted by different superscripted letters differ significantly at the *P* < 0.05 level (*P* < 0.05).

The effects of 7S and sodium butyrate on the distal intestinal gene expression of hybrid grouper are presented in Fig. 5. Compared with the FM group, the mRNA levels of TGF-β1, occludin, claudin3 and ZO-3 were upregulated and IL-1β was downregulated in the bL; the mRNA levels of TNF-α, IL-1β, hepcidin, IL-6, IL-8 were downregulated, and occludin, claudin3, claudin12, claudin15, ZO-1, ZO-2 and ZO-3 were upregulated in the bH. Compared with the bH, TGF-β1, IL-8, jam1, occludin, claudin3, claudin12, claudin15, ZO-1, ZO-2 and ZO-3 mRNA levels were upregulated and TNF-α and IL-6 were downregulated in the bH-NaB. There were no significant difference of IFN-γ and jam4 among the four groups.

**Figure 6:**
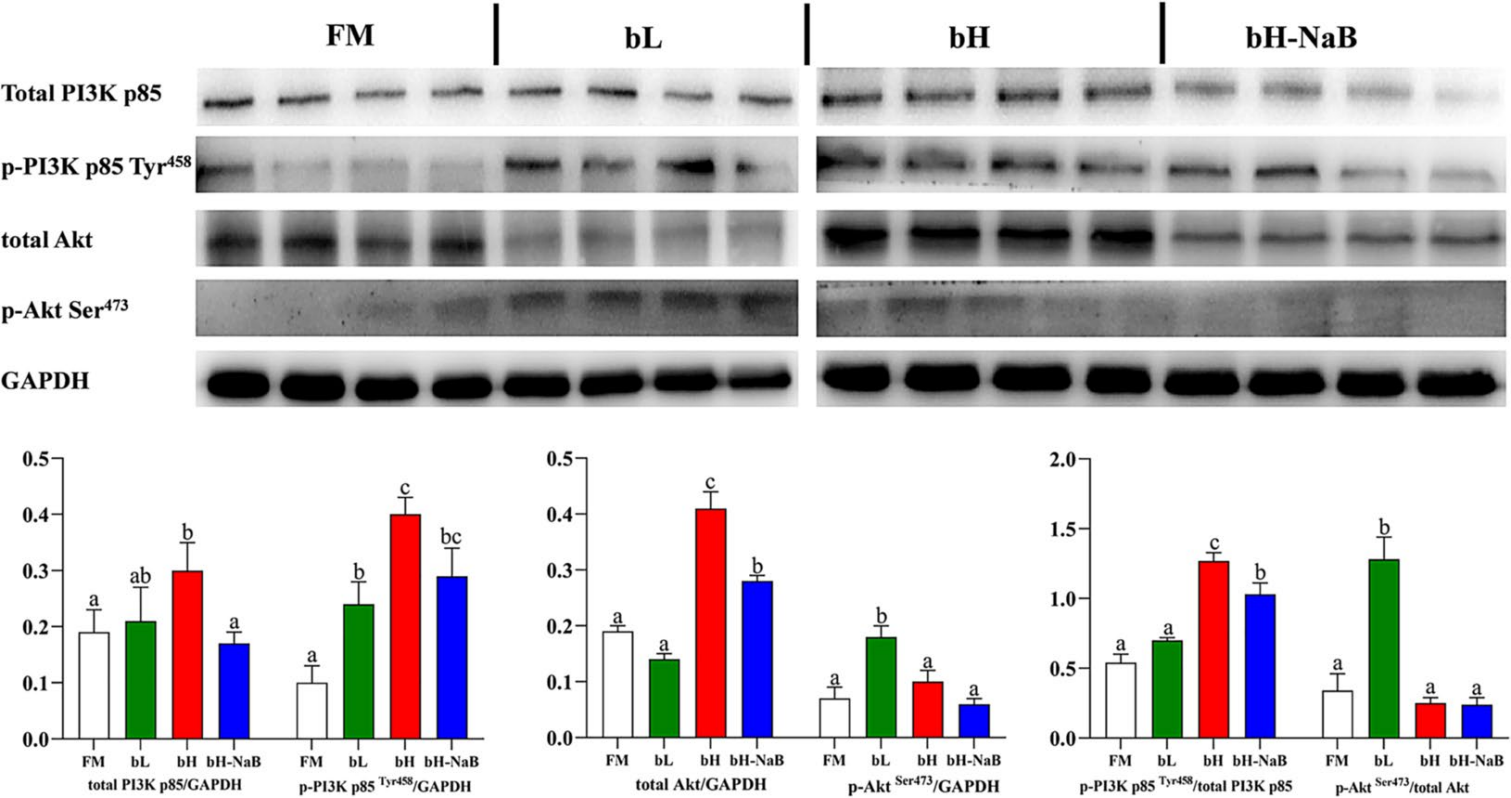
Dietary modulations of distal intestinal responses in the target of PI3K/Akt signaling pathway. The samples derive from the same experiment and that gels and blots were processed in parallel. Results are represented as mean ± SE (n = 4). Significance was evaluated by one-way ANOVA followed by Tukey’s multiple range tests. *FM* control diet, *bL* containing 1.5% 7S diet, *bH* containing 6% 7S diet, *bH-NaB* containing 6% 7S and 0.13% NaB diet. ^a,b,c^Mean values among all treatments with different letters were significantly different when the interaction was significant (*P* < 0.05).

For western blot analysis of PI3K-Akt (Fig. 6), the total PI3K p85/GAPDH value in the bH was significantly higher than those in the FM and bH-NaB, and the p-PI3K p85 ^Tyr458^/total PI3K p85 value in the bH was significantly higher than those in the FM and bL, the p-PI3K p85 ^Tyr458^/total PI3K p85 value of the bH-NaB was lower than that of the bH, although there was no significant difference. The total Akt/GAPDH values in the FM and bL were significantly lower than that in the bH and bH-NaB, sodium butyrate supplementation significantly reduced the total Akt/GAPDH value in the bH-NaB; the p-Akt ^Ser473^/GAPDH values in the bL, bH and bH-NaB were significantly higher than that in the FM. There was no significant difference in p-PI3K p85 ^Tyr458^/total PI3K p85 values between the FM and bL, but both were significantly lower than the bH. After supplementation with sodium butyrate, p-PI3K p85 ^Tyr458^/total PI3K p85 value in the bH-NaB was significantly lower than that in the bH. The p-Akt ^Ser473^/total Akt values in the FM, bH and bH-NaB were significantly lower than that in the bL, and no significant difference was observed in the FM, bH and bH-NaB.

## Discussion

In general, fish intake of incompletely digested 7S usually leads to metabolic disorders and adverse physiological and biochemical reactions, which can have the effect of disrupting the digestion and absorption of nutrients in the intestines^26^. Interestingly, the weight gain was significantly higher in the bL than in the FM, suggesting that 1.5% 7S addition could promote the growth of hybrid grouper, which deserves further in-depth study. In turbot (*Scophthalmus maximus* L.), a reduction in feed utilization efficiency following ingestion of feeds containing 4–8% 7S has been observed to result in a significant reduction in specific growth rate^17^. Similar results were also found in Jian carp (*Cyprinus carpio var*. Jian), where supplementation of 8% 7S in the diet significantly reduced the SGR of Jian carp^4^. These findings were similar to those obtained in the present study, in which we observed that hybrid grouper receiving a diet containing 6% 7S showed a significant reduction in instantaneous growth rate and weight gain.

In the present study, we found that the addition of sodium butyrate significantly increased instantaneous growth rate in the bH-NaB group compared with that in the bH group, indicating that NaB can be effective in ameliorating the growth inhibition effects attributable to high doses of 7S. We also found that the feed coefficient ratio of fish in the bH and bH-NaB groups was significantly higher than that in the FM and bL groups, whereas the addition of NaB significantly reduced the feed coefficient ratio, indicating that butyrate may enhance the growth performance by improving the feed utilization of hybrid grouper, which is similar to results obtained for grass carp^10,27^. We speculate that this effect of sodium butyrate is associated with an increase intestinal antioxidant capacity and enhancement of intestinal immunity.

To further investigate the effects of 7S and sodium butyrate on the disease resistance of the hybrid grouper, we conducted a 7-day challenge test using *Vibrio parahaemolyticus* injected into fish at a concentration of 7.41 × 10^8^ CFU/mL at the end of the breeding experiments. We accordingly observed the highest cumulative mortality in the bH group, thereby indicating that high doses of 7S can significantly reduce disease resistance in hybrid groupers, whereas resistance was enhanced to some extent in groupers fed a diet supplemented with sodium butyrate. Interestingly, whereas the trend in mortality in the bH group showed a consistent and rapid increase during the first 4 days post-injection, the upward trend only became apparent at day 4 post-injection in groupers fed the NaB-supplemented diet. These observations thus tend to indicate that although sodium butyrate may not be effective in ameliorating the reduced disease resistance caused by high doses of 7S, it can, to a certain extent, delay the onset of disease caused by *V. parahaemolyticus*, thereby gaining valuable time for treatment. However, given that it was unclear how this resistance is regulated, we conducted the research described in the following section.

There are many cytokines present in the fish immune system, and most of them could exercise immune regulation and play an important role in maintaining the stability of the internal environment of the organism^28^. As a typical pro-inflammatory cytokine, IL-1β is primarily produced by mononuclear macrophages, and can induce the activation of neutrophils and macrophages, stimulate the release of thromboxane and platelet-activating factor, increase the permeability of epithelial and endothelial cells, and induce intestinal inflammation^29^. In the present study, we found that levels of IL-1β in bL and bH group fish were significantly down- and upregulated, respectively. The lower levels of IL-1β in the bL group conceivably indicate that low-dose 7S has the function of indirectly reducing IL-1β level of serum. Since there are few studies on the effects of 7S on serum IL-1β, the reason why low levels of 7S reduced IL-1β may still need further studies. Whereas the significantly elevated levels of IL-1β in the bH group, could indicate that at higher doses, 7S may exacerbate the systemic inflammatory response by increasing the level of IL-1β. We also observed that sodium butyrate supplementation of the grouper diet had the effect of significantly reducing serum IL-1β, thereby indicating that sodium butyrate may modulate serum immunity by reducing the level of IL-1β. In this regard, studies on rats have found that sodium butyrate is effective in the topical treatment of TNBS (trinitrobenzene sulphonic acid)-induced colitis in mice, and significantly reduced serum IL-1β production^30^. This is consistent with the results of our experiment, and we suspect that a similar regulatory mechanism exists for serum IL-1β in mice and hybrid grouper with sodium butyrate.

TNF-α is a further key cytokine that plays role in mediating intestinal inflammatory processes^31^, and has been shown to exacerbate the progression of enteritis by inducing the release of pro-inflammatory factors^32^. Consequently, determining changes in the levels of TNF-α is important with respect to monitoring inflammatory responses in the body. We found that a low dose of 7S significantly indirectly reduced serum TNF-α levels in the hybrid grouper, whereas in contrast, a high dose had the effect of promoting the level of TNF-α. Similarly, in turbot, it has been found that supplementing feed with 4–8% 7S increased the expression of TNF-α, which ultimately inhibited growth^17^. These observations indicate that 7S might have an indirect effect on TNF-α level in both hybrid grouper and other fish. We also found that NaB significantly downregulated the elevation of serum TNF-α induced by high-level 7S. Similar results also have been observed on the distal intestine of common carp (*Cyprinus carpio*), addition of 0.03% microencapsulated sodium butyrate significantly reduced the transcript level of TNF-α in distal intestine induced by oxidized fish oil^10^. This effect may also be associated with the fact that sodium butyrate inhibits mast cell activation and suppresses the release of mast cell inflammatory mediators^33^.

Similarly to TNF-α, the type II interferon IFN-γ play roles in immune and inflammatory responses^34^. On the basis of the findings of the present study, it would appear that both low and high doses of 7S can increase the level of IFN-γ, and we accordingly speculate that 7S may have an indirect positive regulatory effect on distal intestinal IFN-γ in hybrid grouper. Interestingly, supplementation with sodium butyrate under high-dose 7S conditions further elevated serum IFNγ levels in hybrid grouper. However, although we found significant differences in serum IL-1β, TNF-α and IFN-γ among the different treatment groups. Since there were no studies on the correlation of IL-1β levels with physiological status in fish, especially in hybrid grouper. Whether the significant changes in serum IL-β, TNF-α and IFN-γ could indicate significant changes in serum immunity in hybrid grouper needs to be analyzed in conjunction with the results of further targeted studies.

Nitric oxide (NO), a free radical gas produced from l-arginine via the catalytic activity of nitric oxide synthase (NOS), plays an important role in host defense and inflammatory responses, and can interact with cytokines such as TNF-α and IFN-γ, thereby influencing the course of inflammatory responses^35–37^. Currently, however, there is a degree of controversy as to the mechanism whereby NO is regulated in the intestines^35,38^. In the present study, we found that total NOS showed an elevated trend in the bL group and NO was significantly elevated in the fish in this group. A similar pattern was also observed in groupers in the bH group. Notably in this regard, we also detected significant increases and decreases in the levels of IFN-γ and TNF-α in the bL group, respectively, the former of which can enhance NO secretion by stimulating macrophages and inducing the production of nitric oxide synthase^39^. We speculate that either TNF-α is not involved at low doses of 7S but is involved at high doses, or, alternatively, that TNF-α does not play a role in regulating the macrophage production of NO in the hybrid grouper. In addition, we observed that dietary supplementation with sodium butyrate maintained the significant upregulated production of IFN-γ, and resulted in the recovery of TNF-α to FM group levels. Correspondingly, total NOS levels were significantly reduced, and NO showed a non-significant downward trend. This suggests that distal intestine NO level in hybrid grouper may not be determined by total NOS. However, since there are different isoforms of NOS in fish, which isoform plays a dominant role needs to be further investigated. The ONOO^−^ is produced by the rapid binding of NO to oxygen radicals, which may be an important factor in cell damage, energy consumption, and cell death^40^. We found that levels of ONOO^−^ in the bL group were significantly downregulated, which contrasts with the elevated levels of NO in this group. Our observations tended to indicate that both IFN-γ and IL-1β and TNF-α are involved in the regulation of the NO-binding peroxide-generating step of ONOO^−^ production. And from the analysis just presented, TNF-α and IL-1β are very important in the regulation of ONOO^−^ generation^41^, particularly at high doses of 7S. We also found that sodium butyrate supplementation significantly reduced total NOS levels, which may be related to the inhibition of inducible NOS promoter-dependent transcriptional activity by sodium butyrate^42^. Excess ONOO^−^ can adversely affect tissues primarily by damaging DNA, inactivating enzymes, and degrading mitochondria, and thus the downregulation of ONOO^−^ in the bH-NaB group may be related to the fact that sodium butyrate can enhance the provision of energy to cells^43,44^, thereby preventing the generation of ONOO^−^ and thus preventing its negative effects. Due to the lack of findings on the correlation between the levels of intestinal ONOO^−^, NOS and their physiological status in hybrid grouper. Therefore, whether the magnitude of changes in ONOO^−^ and NOS in the present experimental results would lead to corresponding changes in the physiological status of hybrid grouper needs to be studied in depth.

As previously mentioned, the strong oxidizing properties of ONOO^−^ and its interaction with cytokines such as TNF-α and IL-1β might induce cellular damage and thus disrupt the normal morphology of tissues. To better visualize the effects of NO and ONOO^−^ on the intestinal tract of hybrid grouper, we prepared histomorphological sections of the hindgut, which is particularly susceptible to 7S-related disturbance (Periodic acid Schiff and Alcian Blue staining, AB-PAS).

Intestinal development in fish can often reflect digestive absorption, and in this regard, fold height is typically used as an indicator of intestinal health. However, we would argue that fold height alone does not truly reflect the status of intestinal health. Therefore, as an alternative, we used the ratio of intestinal diameter to fold height (Id/Fh), which we believe to be a more reliable indicator of intestinal integrity. The larger this ratio, the greater is the space occupied by folds in the intestines, and thus the greater is the efficiency of absorption^27^. On the basis of the findings of the present study, we established that a high dose of 7S can significantly increase the Id/Fh ratios in the distal intestine compared with the FM of hybrid grouper, thereby reducing the absorptive area of the intestinal fold and thus the efficiency with which feed nutrients are absorbed. However, supplementing the grouper diet with sodium butyrate reversed the detrimental effects of high-dose 7S, resulting in a reduction in the Id/Fh ratio. We speculate that this may be related to the function of appropriate NaB doses in promoting proliferation and supplying energy to intestinal epithelial cells^45,46^. Although there was still a significant difference in the bHNaB compared with the FM, NaB still had a significant promotion effect on the growth of distal intestinal folds in hybrid grouper. Similar results have also been obtained for the yellow drum (*Nibea albiflora*, Richardson)^47^.

The role of goblet cells in fish is mainly through the regulation of the secretion of mucus and mucus-associated anti-microbial substances, such as antimicrobial peptides^48^, and it is also an important component of the innate immune system. Mucus secreted by goblet cells plays an important role as a barrier in the fish intestine^49^. In the present study, we observed that the numbers of goblet cells in the intestines of bL and bH-NaB group fish were not significantly different from those in the FM, whereas in contrast, numbers were significantly reduced in the bH group, thereby indicating that a high dose of 7S had a significantly negative effect on the number of distal intestinal goblet cells. Conversely, supplementing the grouper diet with sodium butyrate was found to effectively increase the abundance of goblet cells. This result suggests that sodium butyrate may enhance distal intestinal barrier function by promoting mucus secretion from goblet cells in hybrid grouper.

Subsequent to the ingestion of antigenic proteins in feed, most is converted to amino acids and other small molecules. However, owing to the high molecular weight of 7S, a small fraction is not readily broken down and directly enters the lymphatic system via the intestinal mucosal spaces to induce an immune response^50^. When the antigen re-enters the body from the intestines, it will cause intestinal mucosa damage and edema, leading to inflammation. In this regard, although numerous studies have examined the effects of antigenic proteins on the intestines of fish, few have investigated the root cause of the inflammatory effects.

In this study, we found the expressions of TNF-α, IL-1β and IL-8 were significantly upregulated in the bH. In the distal intestine. In turbot, addition of 4–8% of 7S to the feed resulted in significant upregulation of distal intestinal TNF-α and IL-1β at the transcriptional level^17^, which was consistent with the results of this experiment. However, when we supplemented with sodium butyrate, we found that TNF-α decreased, but IL-1β and IL-8 continued to be highly expressed. This may indicate that there are other regulatory factors between TNF-α and IL-1β in regulating distal intestinal inflammation in hybrid grouper, rather than direct regulation. TGF-β1, a typical anti-inflammatory factor, has a very important function in the immune system of fish. TGF-β1 blocked the proliferation of peripheral blood lymphocytes in grass carp induced by phytoagglutinin or lipopolysaccharide stimulation^51^ and had a downregulatory effect on the nitric oxidative response of TNF-α-activated macrophages^52^. Downregulation of TGF-β1 resulted in enhanced sensitization of intraepithelial lymphocytes and failure to maintain normal hindgut mucosal integrity^53^. And we also obtained similar results as for turbot, where the addition of low dose 7S (2%) effectively promoted the expression of TGF-β1, while TGF-β1 transcription was significantly reduced when the addition amount was increased to 4–8%^17^. These results suggest that similar regulatory mechanisms exist for TGF-β1 in turbot and hybrid grouper at low and high doses of 7S. Hepcidin is an important component of innate immunity in fish. It has a wide range of actions and is active against both gram-negative and gram-positive bacteria, with antiviral and immunomodulatory effects^54,55^. We found that only high doses of 7S resulted in a significant increase in hepcidin mRNA level. This is a side effect of the activation of innate immunity in fish by high doses of 7S. In contrast, the expression of hepcidin was not altered under low-dose 7S conditions, indicating that low-dose 7S had no significant effect on the transcription of hepcidin in the distal intestine of hybrid grouper. Supplementation of sodium butyrate in the conventional grass carp formulation effectively promoted the hepcidin transcription in the distal intestine, whereas the expression of hepcidin in the bH and bHNaB in the present experiment was not significantly different from the results of the present experiment^56^. We speculate that this may be caused by different fish species. The host can induce and secrete large amounts of IL-6 protein early in aberrant infections^57^. Therefore, IL-6 is often considered as an acute pro-inflammatory factor and plays a very broad function in immune regulation^58^. Combined with the results from the bHNaB, we can find that sodium butyrate could relieve distal intestinal inflammation by decreasing distal intestinal IL-6 transcription in hybrid grouper.

Intestinal structural integrity is an important basis for vertebrate intestinal health. Tight junction proteins are closely related to the barrier function of the intestine^59^. Changes in intestinal permeability can lead to impaired digestion, which can negatively affect fish growth. Damage to the intestinal barrier function of fish caused by soybean meal-based proteins has been a difficult problem in aquatic animal nutrition^60^. To verify the relationship between this barrier damage and 7S in soybean meal, we further determined the expression of genes related to tight junction proteins in the distal intestine of hybrid grouper. From this experiment, different doses of 7S have no effect on the expression of jam1 and jam4 in the distal intestine of hybrid grouper. Zhang^61^ found no effect on the expression of jam in the distal intestine even when using different levels of soybean meal fed to hybrid grouper, which is consistent with the results of this experiment, indicating that jam1 and jam4 might not be responsible for 7S-induced intestinal inflammation. Interestingly, the up-regulated relative expression level of jam1 in the bH-NaB seems to indicate that the addition of sodium butyrate can further stabilize the barrier function of the intestine through jam1. Occludin, claudin3, claudin12 and claudin15 belongs to transmembrane proteins, and ZO-1, ZO-2 and ZO-3 belongs to cytoplasmic proteins. Transmembrane and cytoplasmic proteins act together to regulate the function of tightly connected barriers. The results of this experiment showed that low and high doses of 7S decreased and increased the tight junction-related gene expressions of hybrid grouper, respectively, and sodium butyrate supplementation significantly upregulated intestinal tight junction-related gene expressions. Claudin3 contributes to the maturation of intestinal barrier function^62^, and is a marker of intestinal injury^63^. Claudin 12 can effectively promote the absorption of Ca^2+^ in the intestine^64^. And Claudin-15 is important for maintaining normal ion permeability selectivity and glucose metabolism in the small intestine of mice^65^. Experimental results show that low dose of 7S could reinforce the intestinal barrier by increasing the expression of occluding and claudin3. We also found that the expression of claudin3, claudin12 and claudin15 was down-regulated at high dose of 7S, while the expression could be up-regulated by supplementation with sodium butyrate. Although we are not sure if this statistically significant alteration would result in physiological changes in hybrid grouper. However, sodium butyrate supplementation significantly increased the transcript levels of claudin3, claudin12 and claudin15, which could be a side response to the fact that sodium butyrate could promote tight junction in the distal intestine of hybrid grouper to some extent. Cytosolic proteins ZOs are a class of peripheral membrane proteins with three isoforms (ZO-1, ZO-2, ZO-3), which can be attached to the intracellular domains of transmembrane proteins occludin, claudins at one end and actin at the other end, thus connecting transmembrane proteins to the intracellular skeletal system and forming a stable tight junctional structure^66^. Among these, ZO-1 is often used as an indicator of tissue tight junction barrier function and permeability. Similar results were also found in piglets^18^. High doses of 7S also inhibited the expression of ZO-1, ZO-2 and ZO-3 in the distal intestine of hybrid grouper, which may also contribute to the increased distal intestinal tight junction. In addition, supplementation with sodium butyrate significantly increased the expression of ZO-1, ZO-2 and ZO-3 in the distal intestine of hybrid grouper, suggesting that NaB has a facilitative effect on the transcription of ZOs.

PI3K/Akt is closely linked to the regulation and release of cytokines in signal transduction pathways^67^. It is able to upregulate the expression of various pro-inflammatory factors by phosphorylating IkBα to activate NF-kB. In this study, high-dose 7S significantly upregulated the expression of p-PI3K/total PI3K, and although the expression of p-Akt/total Akt was not significantly different from that of the FM, total Akt was significantly elevated, suggesting that PI3K/Akt is involved in the inflammatory response induced by high-dose 7S. From the results of the bHNaB, protection and relief of inflammation in the distal intestine of hybrid grouper might be related to the regulation of PI3K/Akt protein expression.

Interestingly, from the results of distal intestinal TUNEL staining, only two apoptotic cells were observed in the field of view of each of the bH and bHNaB. In cholangiocarcinoma cells^68^ and procine small intestinal epithelial cells^69^, inhibition or knockdown of PI3K/Akt can effectively promote apoptosis. Combined with the results of this experiment, we hypothesized that 7S inhibited apoptosis in the hindgut of hybrid grouper while promoting the expression of PI3K/Akt. Alternatively, intestinal apoptosis in hybrid grouper was unaffected by PI3K/Akt. Then how PI3K/Akt acts in the distal of hybrid grouper and executes the immune response needs further in-depth study.

## Conclusion

In the present study, we demonstrated that low and high doses of β-conglycinin (7S) significantly enhanced and reduced the weight gain, instantaneous growth rate, and feed utilization of hybrid grouper, respectively. However, we found that supplementation of the grouper diet with sodium butyrate was effective in relieving the growth inhibition caused by high doses of 7S. High-dose 7S was shown to disrupt the development of distal intestinal fold, suppress the density of distal intestinal goblet cells, and augment the level of NO, NOS, and ONOO^−^ associated with intestinal injury, thereby exacerbating the process of distal intestinal inflammation. The addition of sodium butyrate could properly mitigate oxidative damage to the intestinal tract. Low and high doses of 7S produced different immune responses in the distal intestine of hybrid grouper and affected intestinal tight junction to different degrees. Sodium butyrate could improve the tight junctions in the distal intestine of hybrid grouper by inhibiting pro-inflammatory factors and promoting the expression of anti-inflammatory factors, thereby maintaining a normal barrier. Meanwhile, the abnormal expression of PI3K/Akt may be closely associated with 7S-induced inflammatory response.

## Supplementary Information

Supplementary Information.

## Data Availability

All datasets generated during and/or analyzed during the current study are available from the corresponding author on reasonable request.
