# Supplementary material for: Dietary supplementation of β-conglycinin, with or without sodium butyrate on the growth, immune response and intestinal health of hybrid grouper
Source: Sci Rep. 2021 Aug 27;11:17298. doi: 10.1038/s41598-021-96693-x (PMC8397726; doi:10.1038/s41598-021-96693-x)
Supplement: Supplementary file 1 — Supplementary Information. [file 41598_2021_96693_MOESM1_ESM.docx]

Title Page

Dietary supplementation of β-conglycinin, with or without sodium butyrate on the growth, immune response and intestinal health of hybrid grouper

Bin Yin^1,2,3#^, Hongyu Liu^1,2,3#^, Beiping Tan^1,2,3^*, Xiaohui Dong^1,2,3^, Shuyan Chi^1,2,3^, Qihui Yang^1,2,3^, Shuang Zhang^1,2,3^

**Author Affiliations:**

^1^Laboratory of Aquatic Animal Nutrition and Feed, Fisheries College, Guangdong Ocean University, Zhanjiang 524025, P.R. China

^2^Aquatic Animals Precision Nutrition and High Efficiency Feed Engineering Research Centre of Guangdong Province, Zhanjiang, Guangdong, China

^3^Key Laboratory of Aquatic, Livestock and Poultry Feed Science and Technology in South China, Ministry of Agriculture, Zhanjiang 524025, P.R. China

***Corresponding Author:** Prof. Beiping Tan: [bptan@126.com](mailto:bptan@126.com)

Running title: β-conglycinin, NaB’s effects on grouper

**Table S1** The composition of the experimental diets

|  | Diets | | | |
| --- | --- | --- | --- | --- |
| Ingredients/% | FM | bL | bH | bH-NaB |
| Red fishmeal | 40.00 | 40.00 | 40.00 | 40.00 |
| Casein | 11.54 | 11.54 | 6.84 | 6.84 |
| Gelatin | 2.89 | 2.89 | 1.71 | 1.71 |
| Wheat flour | 20.00 | 20.00 | 20.00 | 20.00 |
| Fish oil | 4.72 | 4.72 | 4.72 | 4.72 |
| Soy lecithin | 2.00 | 2.00 | 2.00 | 2.00 |
| Calcium monophosphate | 1.00 | 1.00 | 1.00 | 1.00 |
| ^a^Vitamin premix | 0.20 | 0.20 | 0.20 | 0.20 |
| ^b^Mineral premix | 0.50 | 0.50 | 0.50 | 0.50 |
| Antioxidants | 0.05 | 0.05 | 0.05 | 0.05 |
| Choline chloride | 0.50 | 0.50 | 0.50 | 0.50 |
| β-Glycinin 7S | 0 | 1.50 | 6.00 | 6.00 |
| Vitamin C | 0.05 | 0.05 | 0.05 | 0.05 |
| Cellulose microcrystalline | 16.55 | 16.50 | 16.34 | 15.01 |
| ^c^Methionine | 0 | 0.02 | 0.09 | 0.09 |
| ^c^Lysine | 0 | 0.00 | 0.00 | 0.00 |
| Microencapsulated NaB | 0 | 0 | 0 | 0.13 |
| Proximate composition (% air dry matter) | | | | |
| ^d^Crude protein | 47.56 | 47.59 | 48.04 | 48.11 |
| ^d^Crude lipid | 10.55 | 10.24 | 10.70 | 10.22 |
| ^d^Moisture | 9.08 | 9.14 | 9.11 | 9.15 |

^a^Vitamin premix (g/kg mixture): vitamin B1, 17.00 g; vitamin B2, 16.67 g; vitamin B6, 33.33 g; vitamin B12, 0.07 g; vitamin K, 3.33 g; vitamin E, 66.00 g; retinyl acetate, 6.67 g; VD, 33.33 g, nicotinic acid, 67.33 g; D-calcium pantothenate, 40.67 g; biotin, 16.67; folic acid, 4.17 g; inositol, 102.04 g; cellulose, 592.72 g. All ingredients were diluted with corn starch to 1 kg.

^b^Mineral premix (mg g-1 mixture):CaCO_3_, 350 g; NaH_2_PO_4_·H_2_O, 200 g; KH_2_PO_4_, 200 g; NaCl, 12 g; MgSO_4_·7H_2_O, 10; FeSO_4_·7H_2_O, 2 g; MnSO_4_·7H_2_O, 2 g; AlCl_3_·6H_2_O, 1 g; CuCl_2_·2H_2_O, 1 g; KF, 1 g; NaMoO_4_·2H_2_O, 0.5 g; NaSeO_3_, 0.4 g; CoCl_2_·6H_2_O, 0.1 g; KI, 0.1g; zeolite powder, 219.9 g. All ingredients were diluted with corn starch to 1 kg. (Obtained from Zhanjiang Yuehua Feed Co. Ltd. , Zhanjiang, China).

^c^Methionine and lysine were added to balance amino acid with control group.

^d^Crude protein, crude lipid and moisture contents were measured value.

**Table S2** Amino acid profile (%) of the diets used in the experiment.

| Essential amino acids | Diets | | | |
| --- | --- | --- | --- | --- |
|  | FM | bL | bH | bH-NaB |
| Methionine | 1.02 | 1.10 | 1.05 | 1.10 |
| Lysine | 3.12 | 3.16 | 3.15 | 3.04 |
| Threonine | 1.77 | 1.74 | 1.76 | 1.73 |
| Isoleucine | 1.78 | 1.88 | 1.84 | 1.79 |
| Histidine | 1.31 | 1.37 | 1.37 | 1.30 |
| Valine | 2.12 | 2.24 | 2.14 | 2.08 |
| Leucine | 3.21 | 3.25 | 3.25 | 3.15 |
| Arginine | 2.28 | 2.44 | 2.57 | 2.49 |
| Phenylalanine | 1.84 | 1.88 | 1.96 | 1.88 |
| Tyrosine | 1.28 | 1.35 | 1.31 | 1.38 |
| Aspartic acid | 3.63 | 3.73 | 4.03 | 3.83 |
| Serine | 1.82 | 1.79 | 1.92 | 1.81 |
| Glutamate | 7.20 | 7.45 | 7.74 | 7.33 |
| Glycine | 2.65 | 2.64 | 2.50 | 2.47 |
| Alanine | 2.42 | 2.45 | 2.44 | 2.41 |
| Cystine | 0.42 | 0.46 | 0.42 | 0.47 |
| Proline | 2.95 | 2.89 | 2.67 | 2.69 |

**Table S3** Primers sequences used for real-time quantitative PCR.

| Name | Sequence (5’-3’) | Product size (bp) |
| --- | --- | --- |
| TNF-α | AACTGTGTGTCCCCACTGCC | 81 |
|  | CCACAGATGGCCCAGGTCAT |  |
| IL-1β | AAGGTGGACGCCAACAGACA | 153 |
|  | GTTCACTGCAGGCTCAGGGA |  |
| TGF-β1 | CTTCTCCTCCTCCTCGCTGC | 195 |
|  | GATGTTGCTGAGGGCTTCGC |  |
| hepcidin | TGTCAATGACCCACTGAGCCTCG | 105 |
|  | TCCACTGCAAACTGCTGGGC |  |
| IL-6 | CAATCCCAGCACCTTCCAC | 84 |
|  | CCTGACAGCCAGACTTCCTCT |  |
| IL-8 | TGTGGCACTCCTGGTTCTCC | 132 |
|  | GGGTTCACCTCCACCTGTCC |  |
| IFN-γ | CGATTCGGTCATCAAGAGCAT | 128 |
|  | CTCCGTCACGACCGACACCA |  |
| jam1 | CACGACAACGATGGCTCACCTC | 98 |
|  | GCATTTCTGAAGGCGGCAATCTTG |  |
| jam4 | CCGACACTACCAAGCCCACAATC | 123 |
|  | GAGCAAAACCAGCAGAGCAAAACC |  |
| occludin | CTGTCACTGTCTATAAGCTACGCTC | 109 |
|  | TCTTAACACTTTGCACATGAAGTGGA |  |
| claudin3 | AAGCAAGGTCAACATGGCGGA | 112 |
|  | GCGCTGCATGTGAAGTGTGATAG |  |
| claudin12 | AGGGATCGCTGTGGCAACG | 97 |
|  | CAGCCCGTCATACACGCTG |  |
| claudin15 | ACTTCAGGACCAGGTCAAAGTTAGG | 112 |
|  | CGATCCAGATTCAGCCAGAGCT |  |
| ZO-1 | TGGAGCTGCGCTTACCTCAC | 108 |
|  | GGTCAATGAGCACAGACACACAGT |  |
| ZO-2 | GTATGGCCTTCGTCTGGGCAG | 98 |
|  | ATATAATGTCCCCTTCCTGCAGGTT |  |
| ZO-3 | TCTCGAGCCTCCTCCAACGC | 121 |
|  | CTTTGTATCTGCTGTCTGGACGGG |  |
| β-actin | TACGAGCTGCCTGACGGACA | 239 |
|  | GGCTGTGATCTCCTTCTGC |  |


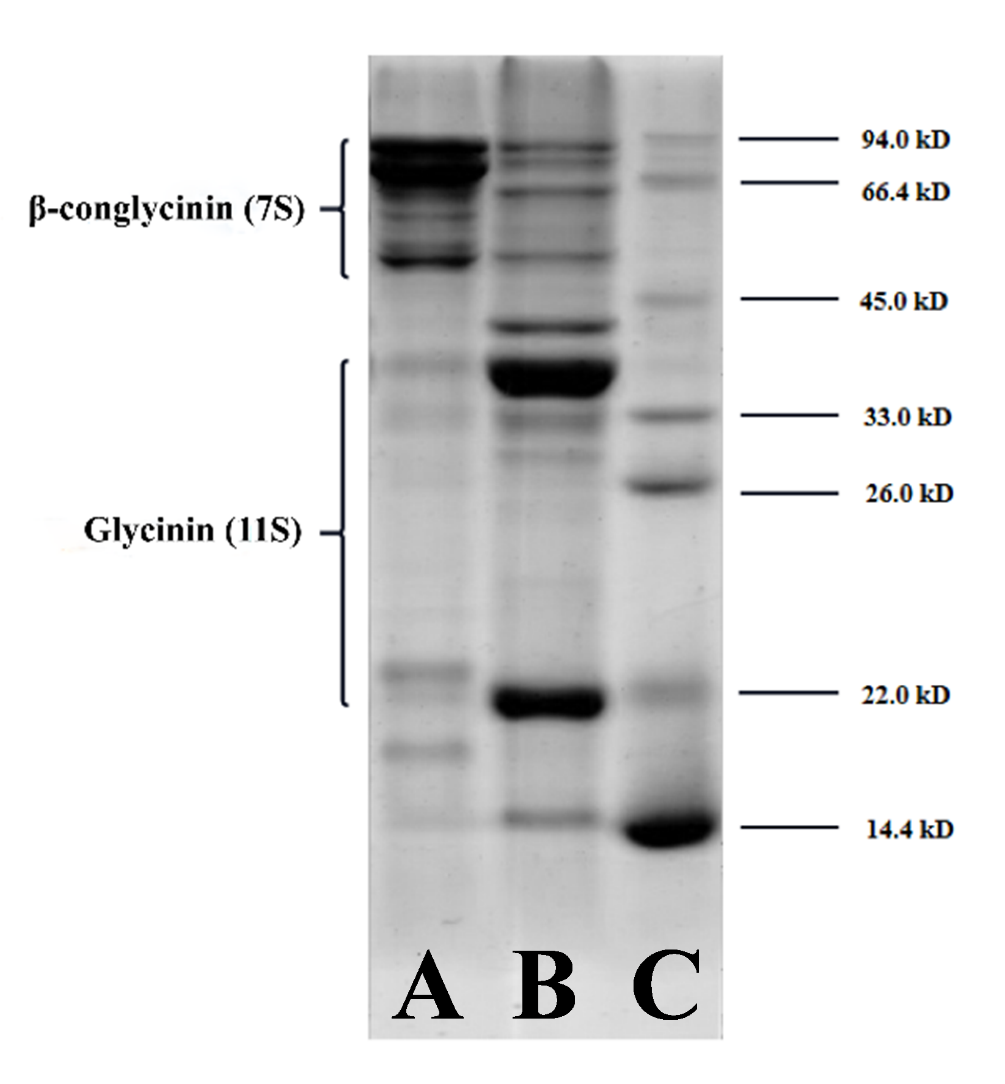


**Figure S1:** SDS-PAGE profile of purified 11S and 7S fraction. Lane A: 7S fraction; Lane B: 11S fraction; Lane C: protein marker.

**Western Blot**

Description:

The western blot results of this experiment were obtained by the traditional method. There are ten holes in one gel. And we use one electrophoresis machine to run two gels for one protein in all samples at the same time, ensuring that the later steps such as transferring, blocking and incubating are performed simultaneously. The samples arrangements are as follow in Example.


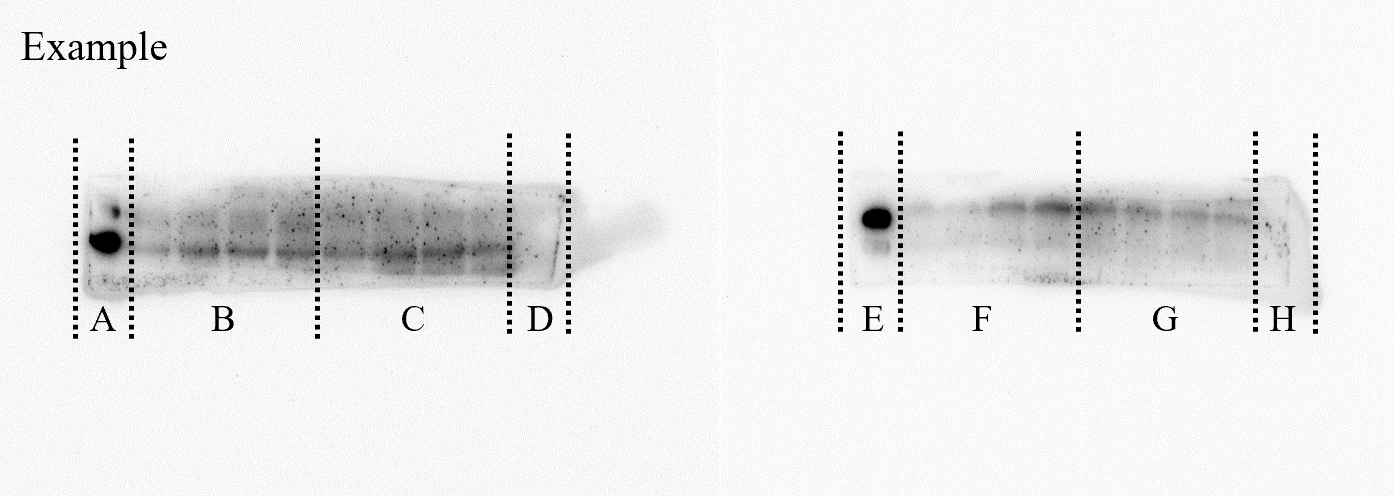
 A: Marker; B: FM group;C: bL group;D: blank (avoid edge effect); E: Marker;F: bH group;G: bH-NaB group; H: blank (avoid edge effect). (n=4)

The original images of each protein produced by chemiluminescence are shown blow.

**total PI3K p85**







**p-PI3K p85 ^Tyr458^**







**total Akt**

**



**

**p-Akt ^Ser473^**







**GAPDH**
